# Functions and Mechanisms of Sleep

**DOI:** 10.3934/Neuroscience.2016.1.67

**Published:** 2016-04-21

**Authors:** Mark R. Zielinski, James T. McKenna, Robert W. McCarley

**Affiliations:** 1 Veterans Affairs Boston Healthcare System, West Roxbury, MA 02132, USA and Harvard Medical School, Department of Psychiatry; 2 Veterans Affairs Boston Healthcare System, Brockton, MA 02301, USA and Harvard Medical School, Department of Psychiatry

**Keywords:** sleep, function, immunity, cognition, energy, EEG, mechanism, neurons, glia

## Abstract

Sleep is a complex physiological process that is regulated globally, regionally, and locally by both cellular and molecular mechanisms. It occurs to some extent in all animals, although sleep expression in lower animals may be co-extensive with rest. Sleep regulation plays an intrinsic part in many behavioral and physiological functions. Currently, all researchers agree there is no single physiological role sleep serves. Nevertheless, it is quite evident that sleep is essential for many vital functions including development, energy conservation, brain waste clearance, modulation of immune responses, cognition, performance, vigilance, disease, and psychological state. This review details the physiological processes involved in sleep regulation and the possible functions that sleep may serve. This description of the brain circuitry, cell types, and molecules involved in sleep regulation is intended to further the reader’s understanding of the functions of sleep.

## 1. Introduction

**Figure 1:**
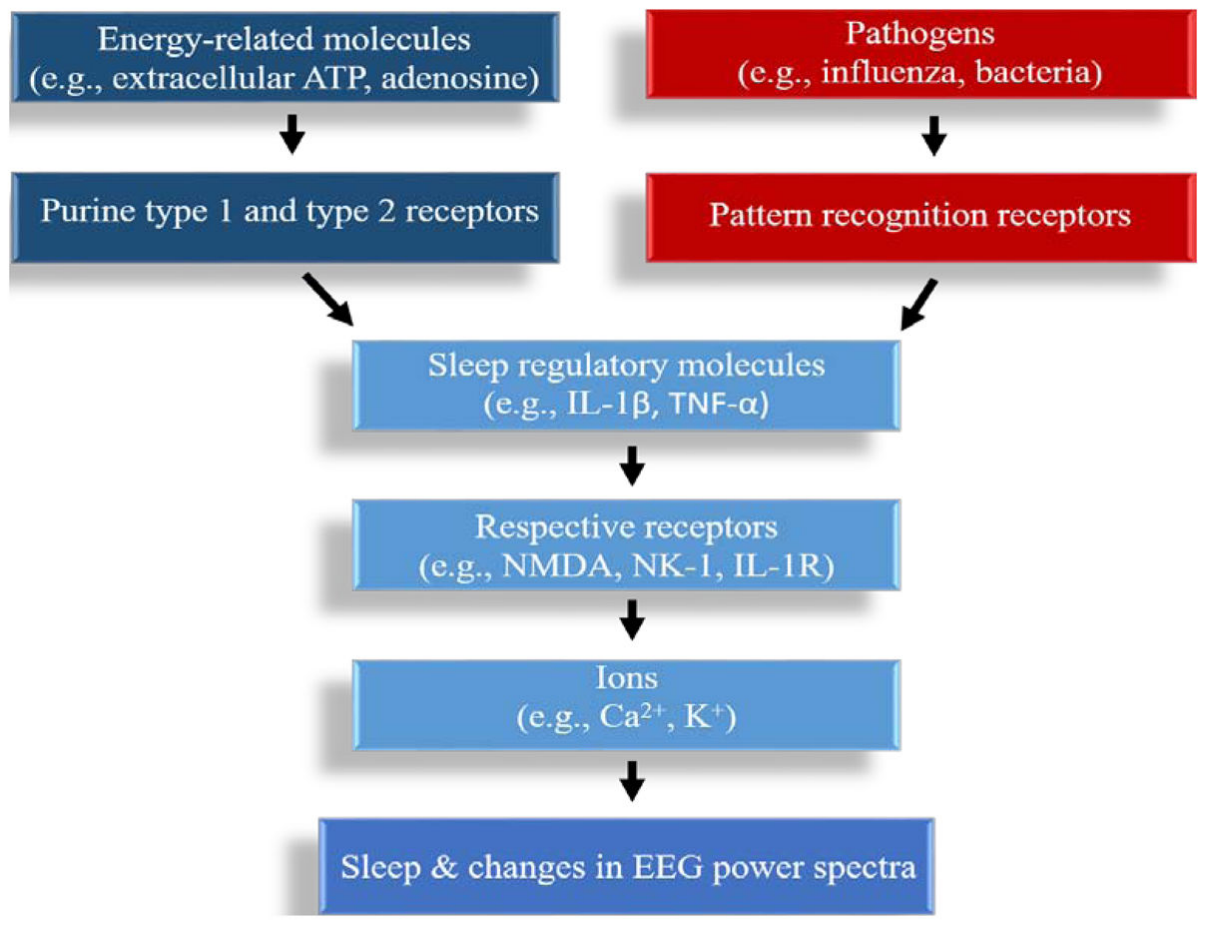
Schematic of sleep regulation Energy-related molecules as well as pathogens through their respective pathogen recognition receptors, are enhanced by waking activity, in turn signaling sleep-regulatory molecules and resultantly promoting sleep. The sleep regulatory molecules act on their respective receptors to signal neurotransmitters. The neurotransmitters alter ion channels, inducing changes in EPSPs and IPSPs and altering sleep and cortical EEG activity.

Sleep occurs in every organism to some extent, indicating its physiological importance [1]. Most sleep researchers agree that a single function of sleep is not a realistic view, as will become evident as this review documents sleep’s essential role in many vital physiologic functions including development, energy conservation, brain waste clearance, and modulation of immune responses, cognition, performance, disease, vigilance, and psychological conditions [2,3]. Sleep has been characterized in many species from humans, birds, fish and flies (e.g., *Drosophila Melanogaster*) to simpler organisms such as worms (e.g., *C. Elegans*) [4]. Sleep may be disadvantageous, for the animal is less vigilant to potential predators, and sleep prohibits consumption of food and procreation. However, animals are not constantly under such pressure. Perhaps, sleep exists, in part, due to the necessity in maintenance of the aforementioned physiological processes at enhanced levels, in order to increase the animal’s ability to survive and propagate. Abundant breakthroughs in our understanding of the basic mechanisms of sleep regulation have occurred within the past 150 years. Nevertheless, sleep is regulated by molecules and pathways that are redundant and also serve other physiological functions, which has made our understanding of the function(s) of sleep and sleep-related pathologies arduous. Herein, we describe how sleep is regulated globally, regionally, and locally, by means of cellular and molecular mechanisms. This description may then serve to suggest how these processes dictate the many functions of sleep (Figure 1).

## 2. Definition of Sleep

The classic definition of sleep is generally based upon physiological characteristics observed in mammals including reduced body movement and electromyographic activity, reduced responsiveness to external stimuli, closed eyes, reduced breathing rates, and altered body position and brain wave architecture assessed by polysomnography (Table 1). However, non-mammalian wakefulness and sleep are often measured by simpler parameters such as decreased relative movement activity, and by rest. Overall, the definition of mammalian sleep relies on activity and metabolism in relation to the electrical brain signals obtained in the electroencephalogram (EEG). Sleep states are typically determined in animals by both the level of muscle activity in the electromyogram (EMG) and EEG characteristics. In humans, sleep states are usually more discernable than in lower animals, including more specific characteristics of EEG architecture. For example, human sleep is classified with 3 defined sub-states of non-rapid eye movement sleep (NREMS) including N1, N2, and N3, which are associated with increasing depth of sleep slower EEG waves, and rapid eye movement sleep (REMS) [5]. Human sleep cycles between NREMS and REMS for approximately 90 minutes for about four to five times during the night in an ultradian cycle [6]. Typically, human sleep is deeper in the beginning of sleep and REMS encompasses a greater proportion of the sleep cycle as sleep persists.

## 3. The Electroencephalogram

Pioneering work demonstrating neural electrical activity provided the foundation of our understanding of sleep states, sleep phenomena, and the molecular mechanisms that regulate sleep [7]. In 1877, Canton recorded electrical impulses from the surface of the cerebral cortex of both rabbits and monkeys using a galvanometer [8]. This finding was independently confirmed by Danilewsky in 1877 [9]. In the 1880s, Ferrier and Yeo examined cortical localization in apes by means of electrical brain stimulation [10,11]. Thereafter in 1890, the physiologist Beck demonstrated that spontaneous rhythmic cortical oscillations in rabbits and dogs were modulated by light, the circadian entrainment signal [12]. Later in 1912, Pravdich-Neminsky reported the first EEG pictorial recordings using a string galvanometer in dogs [13]. He also observed evoked potentials in the cortex of dogs, again suggesting a crucial role of the cerebral cortex in the generation of the EEG. Consequently, this led to detection of EEG recordings in human sleep by Berger in 1924 [14].

**Figure 2:**
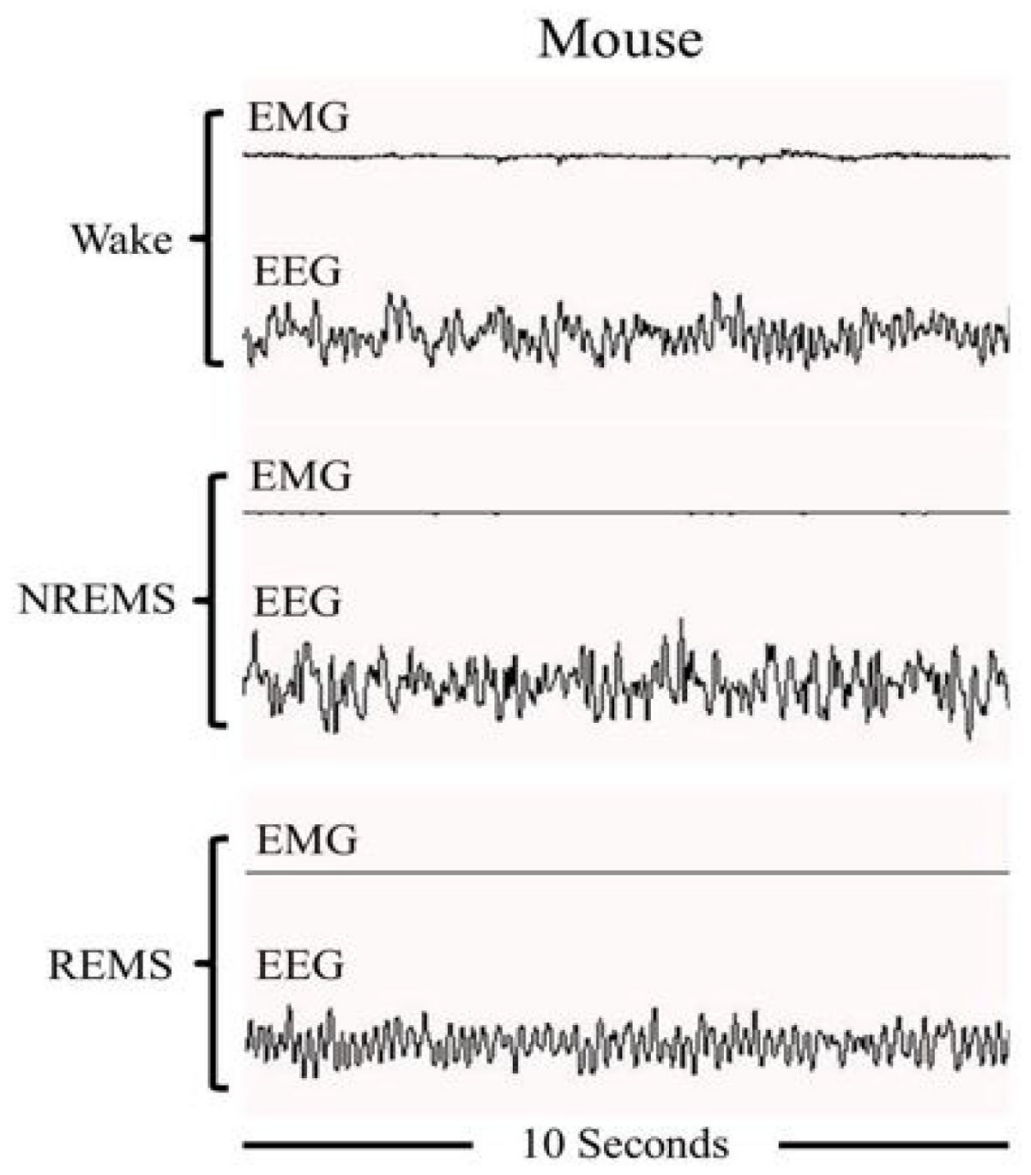
Electroencephalogram (EEG) and electromyogram (EMG) during non-rapid eye movement sleep (NREMS), rapid eye movement sleep(REMS), and waking sleep states in a mouse.

Definition of vigilance states [wakefulness, NREMS, and REMS (described below)] is fundamental to further understand the mechanisms that regulate sleep (Figure 2). In 1953, Kleitman and Aserinsky identified REMS as a unique sleep phenotype characterized by rapid eye fluctuation movement, muscle atonia (with the exception of muscles that control eye movements, the heart, and diaphragm), and a rapid low voltage EEG [15]. The other discernable sleep state was described as NREMS, which is characterized by slow and high voltage EEG, and reduced heart rate and blood pressure. Both NREMS and REMS are found in most mammals including mice, rats, rabbits, dogs, cats, and primates [1]. In 1958, Dement reported a cyclic nature of sleep/wake involving sleep cycle transitions in cats [16]. Jouvet identified REMS as a unique paradoxical sleep state, for EEG recordings were similar to those seen during wake, compared to NREMS that contains slow waves [17]. Subsequently, these findings led to the identification of unique oscillations in the EEG that are characterized by different ranges/bands of signal frequency (Table 2).

## 4. Electroencephalogram Frequency Bands

Electrical brain activity occurs, in part, from ionic current changes within neurons and, to a lesser extent, some types of glia [18–20]. EEG signals are largely the product of synchronized synaptic currents generated by the apical dendrites of pyramidal neurons [21]. Nevertheless, intrinsic membrane properties, neuronal firing, and glial activity likely also contribute to the EEG signals. EEG frequency bands provide essential information of how brain regions, cells, and molecules regulate wakefulness, sleep states, and display dysfunction due to related pathologies (Table 2).

### 4.1. Delta rhythms

EEG delta rhythms are of approximately 0.5–4 Hz frequency range. Delta rhythms are most prominent during NREMS, and are often referred to as slow-wave activity (SWA) when evident during NREMS [21]. Evidence suggests that delta rhythmic activity is produced by hyperpolarization of the intrinsic membrane potential of cortical pyramidal and thalamic relay neurons, for excitatory input to these neuronal populations is withdrawn as sleep is initiated and progresses. Delta rhythms are also present during wakefulness and, to a lesser extent, during REMS. Delta rhythms are also associated with the formation of K-complexes, which are EEG phenomenon that suppress cortical arousal early after NREMS begins. K-complexes are often followed by subsequent sleep spindles, which are bursts of oscillatory EEG activity occurring ~12–14 Hz for < 1 second. Delta rhythms are enhanced after quiet NREMS begins and become progressively stronger as NREMS persists. These waves are enhanced during NREMS after sleep deprivation, including a compensatory negative rebound. SWA exhibits a diurnal rhythm [22]. Moreover, Steriade and colleagues demonstrated in cats a slow (0.5–1 Hz) oscillatory activity originating in the frontal cortex that spreads to more posterior cortices as NREMS progresses [23]. Subsequent studies in both humans and animals have revealed that these oscillations play a role in synchronization of other NREMS-related oscillatory activity, including that in the spindle and delta range [24,25].

NREMS delta rhythm intensity (SWA) is often used as an indicator of sleep intensity. The two-process model of sleep regulation was proposed by Borbély and colleagues, in which both homeostatic (Process S) and circadian (Process C) sleep drives are described [26]. Indeed, the neurobiology of circadian rhythms is a vital component of sleep regulation (described in more detail in the review) [27]. In the mathematical model description of the two-process model, sleep need is described as the intensity of NREMS delta activity [26]. However, Dijk and Czeisler found a disassociation between contributions of circadian rhythms and the homeostatic sleep drive with NREMS SWA in humans [28]. Furthermore, there are many examples where NREMS delta power and sleep drive and sleep duration are regulated independently [29]. Therefore, the use of delta rhythm activity as an indicator of sleep drive should be made with trepidation.

Ground breaking work by Steriade and colleagues revealed that delta rhythms occur in the thalamo-cortical loop [30], although the hypothalamus and basal forebrain also strongly influence cortical NREMS delta activity. The exact mechanism that regulate delta rhythms are currently unknown, although they are associated with changes in cerebral blood flow, as well as modulation of vascular hemodynamics by molecular and cellular activity [31,32].

Delta rhythmic activity during sleep is associated with enhanced subsequent cognitive performance and attention during waking, revealed by investigations employing such methods as sound stimulation and transcranial magnetic stimulation [33,34]. Delta rhythms are prominent in young people, and generally their dominance is reduced with aging [35]. Pathologies involving sleep disturbance including schizophrenia, type II diabetes, and cardiovascular disease are associated with altered delta rhythms [2,36]. Moreover, sleep disorders, including sleep apnea and insomnia, are associated with abnormal delta rhythmic activity.

### 4.2. Theta rhythms

Theta rhythms are found at approximately the 4–9 Hz frequency range in all mammals. Theta rhythms are dominant during REMS [21], particularly when recorded in the hippocampus, and also occur during NREMS, although they are a minor component of the frequency spectra compared to delta rhythms. Typically, theta rhythms become more prominent during transitions between NREMS to REMS. The hippocampus and the medial septum, among other brain regions, are crucial in regulating theta rhythms [37]. However, the hypothalamus and brainstem, including REM-on and REM-off neurons, may also regulate theta rhythms [38].

Theta rhythms are also evident during wake, and theta rhythm enhancements that occur during cognition may play a role in memory consolidation during subsequent REMS bouts [39]. Theta rhythms are also associated with enhanced brain activity related to movement. Abnormalities in theta rhythms also occur in diseases associated with disturbed sleep, such as Alzheimer’s disease and type 2 diabetes [40,41]. Furthermore, individuals with REMS behavior disorder exhibit dysregulated theta rhythms [42].

### 4.3. Alpha Rhythms

Typically, alpha rhythms are found around the 9–15 Hz frequency range. Alpha rhythms are mostly found during quiet wakefulness [21]. During relaxed states when the eyes are closed, alpha rhythms are more pronounced, and are suppressed when the eyes are open. In general, alpha waves are attenuated during relaxed wakefulness, as drowsiness occurs, and during sleep. Alpha rhythms originate from thalamo-cortical networks including the occipital lobe, and are modulated by brainstem cholinergic input [43].

Alpha rhythms are associated with neuronal network coordination and cognition, and are quite prominent in states of coma [21]. Alpha wave intrusion into delta waves is observed in individuals suffering from major depressive disorder—a disorder associated with sleep disturbance—as well as other sleep disorders including insomnia, periodic limb movement disorder, circadian rhythm sleep disorders, sleep apnea, and narcolepsy [44].

### 4.4. Beta Rhythms

Beta rhythms are low amplitude EEG frequencies typically found at the 15–30 Hz frequency range. Beta rhythms are a key component of quiet waking and normal consciousness [45]. Evidence suggests that beta rhythms are important neural regulators that synchronize the activity of spatially distant brain regions. These rhythms are present during heightened states of alertness and critical reasoning, and some groups have proposed that these rhythms may be produced by similar neuronal mechanisms that also govern slower gamma rhythms (see below). Beta rhythms are also involved in anxious thinking and active concentration, and are enhanced during muscle contractions. Beta rhythm dysregulation is associated with pathologies involving sleep dysregulation, including Parkinson’s disease.

### 4.5. Gamma Rhythms

**Figure 3:**
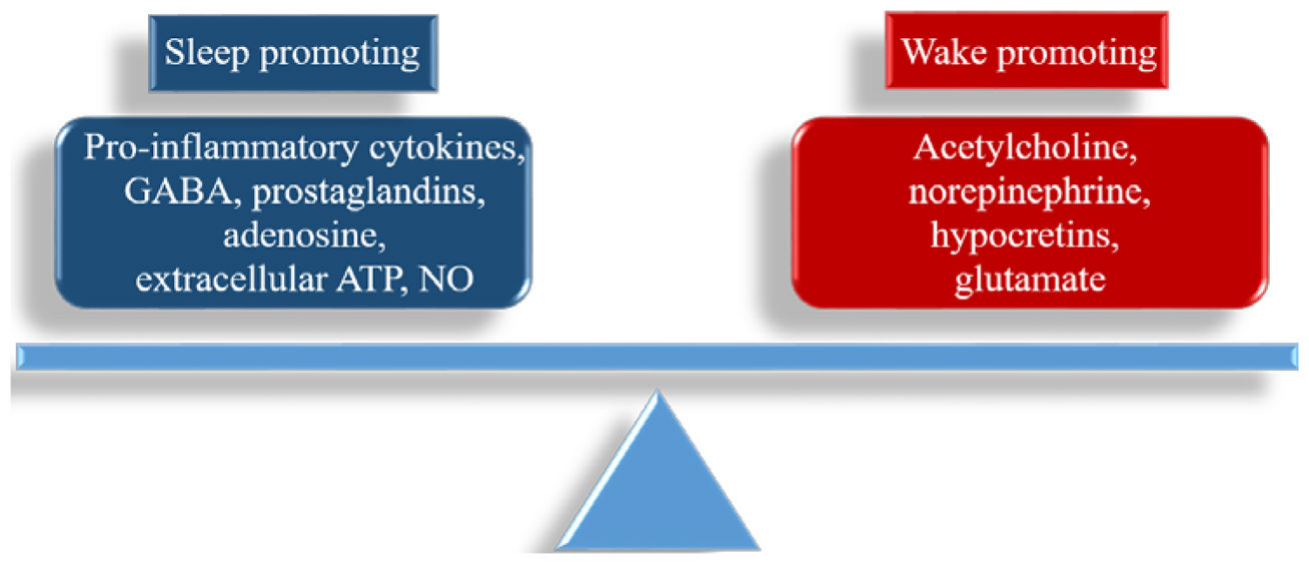
Sleep and wakefulness are determined by a balance of sleep-promoting and wake-promoting substances.

Gamma rhythms are EEG oscillatory activity occurring at approximately 30–120 Hz frequency, and are usually of low amplitude in the cortical EEG. Similar to beta rhythms, gamma rhythms occur during quiet wakefulness and are associated with consciousness, and are enhanced in the cortex after sensory stimuli [21,46]. Gamma rhythms are typically enhanced with theta rhythms during active waking, as well as during REMS particularly in association with **p**onto-**g**eniculo-**o**ccipital (PGO) activity (see below REMS Networks section). Gamma rhythms are also present during the upstate of slow waves during NREMS, and are enhanced after sleep loss. Gamma rhythms are generated in the neocortex by fast-spiking interneurons expressing the neurotransmitter gamma-aminobutyric acid (GABA) and calcium (Ca^2+^) binding protein parvalbumin (Figure 3), which are reciprocally connected to cortical glutamatergic principal neurons. *In vitro* and *in vivo* evidence indicates that firing of these fast-spiking interneurons phase-lock and enhance the synchrony of gamma activity. Recent studies using optogenetics (mice expressing the channelrhodopsin-2 (CHR2) transgene, allowing neurotransmitter phenotype-specific neuronal activation by light) found that excitation of fast-spiking interneurons in the barrel cortex enhanced gamma waves, while inhibition of these cells with optogenetics reduced gamma oscillatory activity [47,48]. The basal forebrain cholinergic system appears to play an important role in modulating gamma activity, by means of excitation of cortically-projecting basal forebrain GABAergic neurons [49]. Of particular importance, McCarley and colleagues reported that basal forebrain GABAergic parvalbumin neurons play a crucial role in the generation of cortical gamma band oscillations [50]. Gamma rhythms are enhanced with signal processing and learning, and rhythmic dysregulation is associated with cognitive difficulty and diseases associated with sleep disturbance, such as schizophrenia [21].

## 5. Sleep-regulating Brain Regions and Networks

Certain physiological functions, such as immune response, development, and cognition, also play a role in regulation of NREMS and REMS, by means of influencing vigilance state-regulating brain regions and networks. For example, research indicates that sleep, especially REMS sleep, is necessary for proper neural development in neonate, termed the ontogenetic REM sleep hypothesis [51]. Sleep loss during development can reduce brain mass, induce neuronal cell death, and increase risk of eventual behavioral problems [52]. One hypothesis of sleep and memory suggests that NREMS and REMS contribute to different types of memory [53]. NREMS appears to be more important for declarative memory whereas REMS is involved more in procedural memory. Others have hypothesized via the sequential hypothesis that both NREMS and REMS work together to enable proper memory functioning. Regardless, animal studies of sleep and sleep loss indicate that proper cognition is dependent upon a complex interaction of brain regions, neuronal and glial interactions, and related molecular mechanisms involving neuroplasticity- and inflammation-related molecules [54]. Recently, Tononi and colleagues proposed the synaptic homeostasis hypothesis (SHY), in which sleep is proposed to selectively attenuate synaptic strength between neurons [55]. Molecular, electrophysiological, and behavioral findings from this group suggest that synaptic strengthening during wake demands energy, and sleep may promote synaptic weakening, ridding the brain of unimportant information, as well as allowing re-establishment of energy reserves and attenuation of cellular stress. Other sleep researchers, though, have argued that there are numerous examples of synaptic plasticity/strengthening occurring during both sleep/wake states and that the SHY hypothesis oversimplifies complex cellular and molecular processes. [56]. Regardless of this dispute, sleep appears to serve a function of re-sculpting the synaptic landscape and energy reserve restoration.

In the early 20^th^ century, von Economo identified brain areas that modulate sleep by studying individuals suffering from “encephalitis lethargica” due to the worldwide flu epidemic in the late 1920s [57]. He concluded that a brain area responsible for sleep induction was located in the anterior hypothalamus, and a wake-promoting region in the posterior hypothalamus. Years later during the 1960s, systematic electrophysiological recordings in neurons within isolated ganglia first recognized neuronal circuits that alter specific behaviors [58]. Thereafter, investigations have mapped the neuronal circuitry that regulates sleep and wakefulness, largely confirming Von Economo’s earlier hypotheses. More recent eloquent and intensive studies have identified excitatory and inhibitory networks that modulate arousal, NREMS, and REMS. Networks that regulate sleep and wakefulness occur, in part, due to neuronal projections to both local and distal brain areas, and substances produced by brain cells may modulate themselves, surrounding cells, and cells in distal brain areas. Overall, there is a balance between arousal-related molecules and sleep-promoting molecules, and this balance may be modulated by brain regions that dominate the production of these molecules, in turn influencing vigilance states (Figure 3).

### 5.1. Arousal Networks

Early work by Moruzzi and Magoun reported that electrical stimulation of the paramedian reticular formation of the midbrain of cats produced EEG desynchronization indicative of arousal/wakefulness [59]. Further investigations demonstrated that arousal/wakefulness is produced by ascending pathways originating in select brainstem monoaminergic and cholinergic neuronal populations located at the mesopontine junction between the pons and the midbrain, and this described neural circuitry is now termed the “ascending reticular activating system” [60]. Brain areas that produce neurotransmitters in this circuitry, especially acetylcholine and monoamines, induce arousal rapidly. The redundancies in arousal molecules and networks likely function to rapidly wake and stimulate an animal to avoid potential danger. However, arousal networks have unique activation characteristics depending upon the stimulus and the prior state of vigilance for particular regulatory brain cells.

Cholinergic neurons in the pedunculopontine (PPT) and laterodorsal tegmental (LDT) nuclei of the brainstem are active during wakefulness, and their output traverses the mesopontine junction to the thalamus, as well as project to the lateral hypothalamus, basal forebrain, prefrontal cortex, and reticular thalamic nuclei [61]. The noradrenergic locus coeruleus (LC), serotonergic dorsal and median raphe, and histaminergic tuberomammillary nuclei all exhibit enhanced activity during arousal [62–64]. These brain nuclei enhance arousal by means of projections to the basal forebrain, lateral hypothalamus, cerebral cortex, and thalamus [65]. In addition, glutamatergic neurons located in the parabrachial nucleus and the adjacent pre-coeruleus area also enhance arousal by means of projections to these upstream regions [66,67].

The basal forebrain and the lateral hypothalamus receive brainstem input, and in turn their ascending projections influence the central neural circuitry involved in modulating arousal [21]. The basal forebrain receives input from brainstem glutamatergic, GABAergic, noradrenergic, serotonergic, and histaminergic neurons, and may in turn influence the thalamo-cortical circuit that promotes arousal [68–72]. GABAergic, glutamatergic, and cholinergic neurons in the basal forebrain directly and indirectly activate cortical pyramidal cells and enhance cortical activation, producing the EEG desynchronization indicative of arousal [50,73,74]. Additionally, the basal forebrain inhibits cortical GABAergic interneurons and deep layer pyramidal cells, thus promoting wakefulness [52,75,76]. The posterior region of the lateral hypothalamus contains neurons that produce hypocretin (orexin) [65]. These neurons project to the entire cerebral cortex, brainstem, basal forebrain, TMN, and LC, as well as to the intralaminar and anteroventral thalamic nuclei, and have been shown to be wake-active. The cerebral cortex, particularly the prefrontal cortex, sends descending innervation back to the basal forebrain, hypothalamus, and brainstem.

### 5.2. NREMS Networks

The lateral hypothalamus, dorsal raphe nucleus, periaqueductal gray, and LC contain high concentrations of GABAergic neurons that are activated during NREMS [65]. Lesion experiments in cats and rats indicate that the hypothalamus and basal forebrain influence NREMS [67,76]. In particular, GABAergic neurons in the ventrolateral preoptic nucleus (VLPO) of the lateral hypothalamus play a significant role in promotion of NREMS [78,79]. VLPO neurons are inhibited by wake-promoting neurotransmitters including acetylcholine, norepinephrine, dopamine, and serotonin. Further investigations have described NREMS-active cell firing in other hypothalamic regions neighboring VLPO, including the median preoptic nucleus [80,81]. The VLPO also innervates LC, raphe system, periaqueductal gray matter, parabrachial nucleus, and histaminergic cells in the TMN, and may inhibit arousal by means of these pathways. In addition, melanin concentrating hormone containing neurons that are present in the cerebellum, lateral hypothalamus, and zona incerta were recently found to regulate both NREMS and REMS [82–84], which might occur through GABAergic release.

### 5.3. REMS Networks

Physiological characteristics of REMS include predominantly fast frequency, low amplitude cortical EEG, hippocampal theta rhythmicity, muscle atonia, PGO-waves, and rapid eye movements. Early investigations reported that lesions to the pons of the brainstem disrupted REMS [85]. McCarley and Hobson then proposed the reciprocal interaction model of REMS control, in which REMS is regulated by interactions of cholinergic, glutamatergic, and monoaminergic brainstem nuclei [86]. The limit cycle model was later proposed by McCarley and Massaquoi [87], in which GABAergic and circadian influences were incorporated into the reciprocal interaction model. REMS is also regulated, in part, by the sublaterodorsal tegmental nucleus and pre-locus coeruleus regions of the brainstem, as well as the medial parabrachial nucleus, which receive GABAergic input from both the ventrolateral periaqueductal gray located within the midbrain and lateral pontine tegmentum of the pons [38,88]. The VLPO is also active during REMS, and VLPO lesions inhibit both NREMS and REMS [65]. Moreover, the ventrolateral periaqueductal gray, sub-lateral dorsal nucleus, and lateral pontine tegmentum may modulate transitions between NREMS and REMS, possibly by means of inhibition of VLPO neuronal activity. Other neural activity, including that of vasoactive intestinal peptide (VIP) neurons located in the suprachiasmatic nucleus within the hypothalamus, may enhance REMS [88].

PGO waves recorded in the cat occur just prior to REMS, generated by the **p**ons, lateral **g**eniculate nucleus of the thalamus, and **o**ccipital cortices [65]. PGO waves first occur during the transition from NREMS to REMS, and then continue throughout REMS, correlating with rapid eye movements. The equivalent to PGO waves in rodent models are termed “P-waves”, for thalamic involvement in wave generation has not been shown. Neuroimaging and electrophysiological studies in humans have suggested that PGO-like cortical field potentials are present during REM sleep [90,91]. Pontine PGO wave-generation is largely due to activity in the cholino-receptive pontine nucleus [92], and PGO waves are synchronized with burst-firing neural activity recorded in cholinergic neurons of the PPT and LDT [93,94].

Saper and colleagues developed the “flip-flop switch” model of REMS, which describes how the induction and inhibition of REMS occurs through a balance of REM-on and REM-off neurons [62]. GABAergic tegmental and periaqueductal gray REM-off neurons inhibit glutamatergic REM-on cells of the parabrachial nucleus (PB) at the junction of the midbrain and pons, as well as the pre-coeruleus (PC). These two nuclei in turn project to the brainstem and spinal inhibitory systems, acting to hyperpolarize motor neurons and induce muscle atonia. During REM sleep, VLPO GABAergic REM-on output reciprocally inhibits GABAergic tegmental/periaqueductal grey REM-off neurons. Glutamatergic REM-on PB/PC neurons send axonal projections to the basal forebrain, thalamus, and cortex, which may modulate both the cortical EEG and hippocampal theta activity. The lateral and posterior hypothalamus also modulate REM-on neurons expressing melanin concentrating hormone. Neurons in the LC, dorsal raphe nucleus, and TNM are mostly inhibited during REMS, further supporting this proposition of select populations of REM-on and REM-off neurons [86,95–97].

## 6. Ionic Regulation of Sleep

Changes in brain electrical activity, reflected in altered EEG frequency bands, are mediated by molecules that influence ion concentrations, such as potassium (K^+^) and Ca^2+^. Ions possess electrical charges that determine the ability of neurons to signal themselves and other cells [98]. The opening and closing of ion channels allow ions to pass through the cell’s plasma membrane, which in turn modulate the membrane potential and, consequentially, may produce action potentials. Ion channel kinetics are regulated by several mechanisms including phosphorylation and dephosphorylation of the protein, the coupling to a messenger molecule such as a neurotransmitter (ionotropic receptors), and by a conformational change in the membrane potential (i.e., voltage-sensitive channels) [99]. Excitatory post-synaptic potentials (EPSPs) occur due to the temporary depolarization of postsynaptic membrane potentials induced by positively-charged ions entering the postsynaptic cell from an opening of their ionic channel. Inhibitory postsynaptic potentials (IPSPs) occur due to negative ionic cellular influx, including chloride, or positively-charged ion efflux. When the postsynaptic potential evokes an action potential it is excitatory, which is typically induced by presynaptic neurons releasing neurotransmitters. The summation of the EPSP and IPSPs of groups of neurons results in hyperpolarized (i.e., down-states) and more depolarized (i.e., up-states) neurons, in turn affecting the EEG and sleep [100].

### 6.1. Potassium channels

Most cell types possess K^+^ channels, and these channels are found in all animals for which sleep is evident [101]. The *Shaker* gene is part of a voltage-gated K^+^ channel that regulates sleep/wakefulness [102]. Flies (*Drosophila melanogaster*) lacking the *Shaker* gene have attenuated sleep and lack sleep responses after sleep deprivation, compared to non-mutant flies. Flies lacking the *SLEEPLESS* gene (a glycosylphosphatidyl-inositol-anchored protein that regulates the *Shaker* gene) or *Hyperkinetic* (a regulatory beta subunit of the *Shaker* K^+^ channel) exhibit reduced sleep [103,104]. Mice that lack the Kv1.2 gene of the Kcna2 channel (a K^+^ channel similar to the *Shaker* channel in flies) also have attenuated NREMS [105]. Another potassium channel, Kv3.1, is present in GABAergic cells that express the Ca^2+^ binding protein parvalbumin, notably in fast spiking neurons. Mice lacking the Kv3.1 channel exhibit attenuated delta oscillations and sleep compared to wild-type mice, further indicating that K^+^ channels play a role in sleep regulation [106,107].

### 6.2. Calcium channels

Pharmacologic and genetic studies provide evidence that Ca^2+^ channels are also involved in sleep regulation [21]. The strongest evidence that Ca^2+^ channels play a role in sleep regulation is provided in studies involving (transient-type) T-type Ca^2+^ channels, but evidence also suggests that L-type Ca^2+^ channels may also modulate sleep [108,109]. Mice lacking the CaV3.1 subunit of the T-type Ca^2+^ channel have reduced NREMS [109]. In addition, another study using Cre/loxP recombination deletion of the gene encoding Cav3.1 subunit of T-type Ca^2+^ channels in the rostral-midline of the thalamus reported reduction of sleep in mice [110]. Mice that lack the alpha1G-subunit of T-type Ca^2+^ channels exhibit a loss of thalamic delta power and sleep spindles [111]. Also, mice lacking both the Ca^2+^ dependent small conductance type K (SK2, Kcnn2) channel and T-type Ca^2+^ channel have reduced NREMS and SWA. Kcnn2 knockout mice also have reduced REMS theta rhythms. A recent study using neuronal ensemble recordings, microdialysis (a sampling technique for the continuous measurement of analyte concentrations from extracellular fluid in tissue), and optogenetic inhibition of thalamic output to the neocortex indicated that NREMS EEG power in the 0.75–1.5 Hz frequency range occurs when T-type Ca^2+^ channels are active, again suggesting that Ca^2+^channels play a role in SWA regulation [112].

## 7. Neurotransmitters

Cajal first revealed the structure of synapses in his histological work over 100 years ago, and speculated that neurons release signaling molecules that are involved in physiological functions [113]. Neurotransmitters that tend to be excitatory, such as acetylcholine, dopamine, norepinephrine, histamine, serotonin, hypocretins (also known as orexin), neuropeptide S, and glutamate, typically enhance arousal or wakefulness [21]. Arousal-related cholinergic and monoaminergic neurons have been extensively investigated though pharmacologic, genetic, and lesion studies, as well as the recent use of transgenic models.

In 1998, De Lecea, Killduff and colleagues recognized hypocretin (orexin) as a sleep regulatory molecule located in the hypothalamus [114]. Independently, Sakurai and colleagues found that hypocretins in the hypothalamus regulate feeding behavior [115]. Hypocretins stimulate and maintain wakefulness, in part, through stimulating the release of wake-promoting neurotransmitters including norepinephrine, dopamine, acetylcholine, and histamine [116]. Hypocretins activate the G-protein coupled receptors hypocretin receptor 1 and hypocretin receptor 2 (also known as orexin 1 receptor and orexin 2 receptor). The hypocretin receptors are activated, in part, through phospholipase C and Ca^2+^-dependent and Ca^2+^ independent pathways to activate protein kinase C, protein kinase A, and mitogen-activated protein kinase (MAPK) signaling pathways, all of which are inflammatory and metabolic pathways that affect sleep/wakefulness. Enhanced wakefulness activates the hypocretin 1 receptor, further supporting the role of hypocretins in promoting wakefulness. Moreover, hypocretin antagonists inhibit arousal. Neurons that produce hypocretins also co-express multiple receptors including glutamatergic receptors [117], adenosinergic A1 receptors [118], muscarinic M3 receptors [119], and serotonergic 5-HT1A receptors [120], allowing hypocretin the ability to rapidly induce changes in sleep/wake states. Many studies have described dysfunction of hypocretin and its receptors in the sleep disorder narcolepsy, again suggesting its unique role in vigilance state regulation [21].

GABA is one of the well-characterized neurotransmitters found to induce sleep and SWA, which occurs, in part, through its ability to modulate the neuronal release of excitatory neurotransmitters including glutamate, acetylcholine, norepinephrine, and hypocretin [21]. GABA functions to largely inhibit the activity of glutamatergic neurons and their respective receptors, thus enhancing NREMS. Glutamate is present in most neurons and, acting as an excitatory neurotransmitter through either the α-amino-3-hydroxy-5-methyl-4-isoxazolepropionic acid (AMPA) or N-methyl-D-aspartate (NMDA) receptor, predominantly promotes arousal and inhibits sleep. GABA acts on both ligand-gated ion channel complex GABA-A receptors and G protein-coupled GABA-B receptors. Both GABA-A and GABA-B receptor antagonists enhance wakefulness while GABAergic receptor agonists promote NREMS. Moreover, well-known GABA-A receptor agonists, including benzodiazepines, barbiturates, imidazopyridines, and cyclopyrrolones, enhance NREMS [121].

Substance P is a neurotransmitter that regulates SWA and possibly sleep duration [122]. Substance P was first discovered by von Euler and Gaddum in 1931, and serves multiple functions including the regulation of mood, anxiety, stress, neurogenesis, nausea, pain, vasohemodynamics and inflammation [123,124]. Substance P release is well-known to induce cytokines, such as interleukin (IL)-1beta (IL-1β) and tumor necrosis factor-alpha (TNF-α), that enhance sleep duration and SWA [2,124]. Recently, neurokinin-1 (NK-1) receptors were found to be co-expressed on cortical sleep-active neurons that express neuronal nitric oxide synthase (nNOS), whose activity is positively correlated with changes in SWA [125,126]. Moreover, injections of substance P fragment 1, 7 enhance SWA locally in the cortical hemisphere where the substance was applied, and NK-1 receptor antagonists attenuated SWA locally, indicating that substance P and the NK -1 receptor regulate SWA [122].

## 8. Humoral Factors and Immune Function

A wide literature indicates that enhanced waking activity occurring during prolonged wakefulness modulates the immune system [2]. In fact, the sleep status of an animal can affect the ability of an animal to respond to infection and wound healing. Furthermore, impaired sleep is evident in diseases that involve enhanced inflammation, including cancer and type 2 diabetes. Pioneering work done by Ishimori and Pieron at the turn of the 20^th^ century led to an understanding of how humoral factors regulate sleep [127,128]. These researchers independently discovered that dogs injected with cerebral spinal fluid from sleep-deprived dogs exhibited enhanced sleep amounts. Their findings led to a search for a single molecule regulating sleep that was termed sleep promoting factor S. At Harvard University, Pappenheimer, Krueger, and colleagues identified factor S in the brain and urine of rabbits and cats as muramyl peptide—a peptidoglycan component of bacteria [129,130]. This led to the identification of numerous humoral factors including cytokines and hormones, which are activated by waking activity and pathogens, regulating sleep. These sleep-promoting humoral factors are activated, in part, through their respective pattern recognition receptors, thereby providing a link between the immune system and sleep regulation.

### 8.1. Cytokines

Cytokines are protein, glycoprotein, or peptide cell signaling molecules that are found throughout the central nervous system (CNS) and periphery that function in autocrine, paracrine, or endocrine fashion [2]. Cytokines play a role in regulation of cognition, performance, appetite, pain, fatigue, sleepiness, sleep, and vasohemodynamics. Cytokine dysregulation occurs in much pathology that involve sleep disturbance, including type 2 diabetes [131], cardiovascular disease [132], and cancer [133]. Cytokine dysregulation is implicated in most sleep-related pathologies including sleep apnea and insomnia [134, 135].

Cytokines function at extremely low concentrations (including femtomolar), and trigger signaling cascades which quickly enhance their actions [2]. Consequently, cytokines are well served to induce changes or detect changes within small regions of the brain and in turn affect the entire CNS to regulate sleep/wakefulness. Many cell types produce cytokines including immune cells, neurons, and glia. Typically, cytokines that are pro-inflammatory, such as interleukin (IL)-1beta (β), TNF-α, IL-18, and IL-6, have NREMS promoting effects [2,136]. Cytokines that have anti-inflammatory properties, including IL-10, IL-13, and tumor growth factor-beta, have anti-somnogenic actions in response to pro-somnogenic stimuli, and likely function to inhibit unbridled inflammation, resultantly preventing brain damage [137–140]. Many cytokines have been identified that alter sleep [2]; however, the most extensively studied cytokines that regulate sleep are the pro-inflammatory molecules IL-1β and TNF-α.

IL-1β and TNF-α exhibit a diurnal rhythm of enhanced expression in the cortex, hypothalamus, and hippocampus, corresponding with changes in sleep propensity in rats [141]. Increased waking activity via acute sleep deprivation enhances IL-1β and TNF-α mRNA expression in various sleep regulatory brain areas including the cortex, hypothalamus, hippocampus, and mesencephalon/pons in rats and/or mice [142–145]. Further, mice injected with the gram negative bacterial cell wall component lipopolysaccharide (LPS) or inoculated with influenza virus exhibit enhanced NREMS and SWA, as well as enhanced IL-1β and TNF-α expression in sleep regulatory brain areas [146–148]. Central and peripheral injections of IL-1β or TNF-α enhance NREMS in rabbits, rats, and mice, as well as alter SWA [2]. IL-1β also attenuates glutamatergic neurotransmission, further suggesting linkage between inflammation and sleep-regulatory cell types [136]. In addition, IL-1β and TNF-α also influence circadian gene expression, such as CLOCK-BMAL1 activation of E-box regulatory element, again potentially modulating sleep/wakefulness [149,150].

Inhibition of IL-1β or TNF-α or their receptors with such methods as pharmaceutical administration, siRNA treatment, or knockout in genetic mouse models produces attenuated sleep responses to sleep deprivation or somnogenic stimuli [2]. In rats, IL-1β and TNF-α gene expression are also enhanced after chronic sleep loss [151]. IL-1β receptors, IL-1 type 1 and IL-1 type 2, are present throughout the CNS including neurons and glia. An IL-1 receptor antagonist inhibits IL-1β from binding to its type 1 and type 2 receptors and has anti-inflammatory actions. Intraperitoneal or intravenous injections of IL-1RA inhibit IL-1β induced NREMS in rats and rabbits. Additionally, mice lacking the IL-1R accessory protein that is specific to the brain (Acpb)(i.e., AcPb inhibits the inhibitor of the IL-1 receptor) have enhanced sleep responses to the gram-negative bacterial cell wall component LPS [144]. TNF-α binds to both TNF receptors 1 and 2 [2]. TNF receptor 1 knockout mice do not have enhanced sleep responses to TNF-α but exhibit enhanced NREMS responses to IL-1β [152]. Mice lacking both TNF receptor 1 and TNF receptor 2 have attenuated NREMS and SWA responses to influenza [148]. Thus, cytokines and their respective receptors play an important role in sleep and SWA regulation, although the complex mechanisms of their activity are not completely known.

### 8.2. Cyclooxygenases and prostaglandins

Cyclooxygenase (COX)-1 and COX-2 are constitutive and inducible enzymes that are found throughout the CNS and periphery, and their functions include modulation of inflammation and sleep [153]. Many sleep enhancing molecules, including IL-1β and TNF-α, increase COX-2 expression. Inhibition of COX-2 attenuates NREMS induced by TNF-α in rabbits [154]. In addition, acetaminophen, which inhibits both COX-1 and COX-2, inhibits the sleep-promoting effects of the bacterial cell wall component muramyl dipeptide [155].

COX converts arachidonic acid to prostaglandin (PG) H2, by PG D synthase, which is the rate-limiting step for the production of PG [153]. Prostaglandins, including PG D2 and PG E2, act through G-protein coupled receptors to modulate inflammation, Ca^2+^ movement, fever, vasohemodynamics, and sleep. PG D2 enhances NREMS when injected into the hypothalamic preoptic region of rats [156]. Mice lacking PG D synthase have normal spontaneous sleep but no NREMS rebound after sleep deprivation [157,158]. PGE2/PG D 2 ratios are enhanced in the pituitary, hypothalamus, and hippocampus of rats after REMS deprivation [159]. Conversely, PG E2 augments wakefulness [160–162]. Evidence also suggests that PG D2 promotes sleep through the PG EP4 receptor, while the waking effects of PGE2 occur through both PG EP1 and PG EP2 receptors [163].

## 9. Glial Regulation of Sleep

Clear evidence indicates that neurons, particularly pyramidal neurons, are important regulators of sleep and EEG activity due to the role they play as the primary electrical conducting cells in the brain [164]. Recent evidence, however, indicates that glia, the most prevalent cell type in the brain, including microglia, astrocytes, and oligodendrocytes, may also play a substantial role in sleep regulation. Glia can express sleep-regulatory neurotransmitters and their respective receptors including GABA and glutamate [18]. Furthermore, evidence indicates that astrocytes and oligodendrocytes can produce action potentials mediated by voltage-gated outward K^+^ currents. Moreover, both neurons and glia are key producers of sleep regulatory humoral factors including cytokines and energy-related molecules, including adenosine tri-phosphate (ATP) and adenosine (see Metabolism and Sleep section below).

### 9.1. Astrocytes

Astrocytes are the most abundant glial cell in the CNS [18]. Astrocytes have many functions including nervous system repair, regulation of ion concentration in the extracellular space, modulation of synaptic transmission, alteration of vasohemodynamics, and as well play a role in metabolism, long-term potentiation, and sleep regulation. Astrocytes produce neurotransmitters including glutamate and GABA, which are co-released with ATP into the extracellular space. Astrocytes can propagate intracellular Ca^2+^ waves over long distances and influence neuronal activity, in part, though neurotransmitter release and sleep regulatory molecules [165]. Within the thalamus, astrocytes exhibit spontaneous intracellular Ca^2+^ oscillations within low frequencies of slow waves (< 0.1 Hz) that can provoke NMDA currents in surrounding neurons *in situ*, suggesting that astrocytes may modulate EEG power [166]. Further, in cats, astrocytic membrane polarization and capacitance oscillations occur in phase with SWA in cortical astrocyte and neuronal recordings [167]. Mice that express the dominant negative SNARE (dnSNARE) transgene, allowing prevention of vesicular release from astrocytes, have impaired EEG architecture, attenuated homeostatic sleep, and diminished SWA response to either sleep loss or LPS administration [168,169]. Moreover, IL-1β levels in astrocytes are highest during times of enhanced sleep propensity, further suggesting that astrocytes are involved in sleep/wake regulation [170].

### 9.2. Microglia and oligodendrocytes

Microglia is found throughout the brain, and their functions include phagocytosis, antigen presentation, repair, extracellular signaling, modulating vasohemodynamics and inflammation, and sleep regulation [2,171]. Minocycline is a drug that inhibits microglia production of cytokines and nitric oxide. Minocycline injections into the peritoneum of mice inhibit spontaneous NREMS implicating microglia in sleep regulation [172]. A specific type of microglia cell, perivascular microglia, are located around the vasculature, and are well positioned to react to cellular activity fluctuations, in turn producing large cascades of highly potent cytokines that may quickly affect other sleep-regulatory cells. Another type of glia, oligodendrocytes, produce a myelin sheath (a multi-layered membrane allowing nerves to produce impulses rapidly) around neurons [173]. Recent evidence indicates that oligodendrocytes are neurotrophic and can modulate sleep, in part, by enhancing both oligodendrocyte precursor cells genes during sleep, as well as oligodendrocyte precursor cell differentiation genes during wakefulness [174].

## 10. Metabolism and Sleep

In 1995, Benington and Heller hypothesized that metabolism is involved in sleep regulation, and sleep in turn serves a restorative function [175]. Many genes involved in regulating metabolism are enhanced during sleep compared to wakefulness, in agreement with this hypothesis [176]. Protein synthesis is generally enhanced during sleep vs. wakefulness, supporting the notion that sleep plays a restorative role. Furthermore, sleep deprivation enhances brain mRNAs that modulate glycogen metabolism and glycogen synthesis [177,178]. Additionally, investigations of energy substrates and their derivatives provide further convincing evidence that metabolic molecules and pathways are involved in the initiation and regulation of vigilance states [2].

### 10.1. Adenosine tri-phosphate

Adenosine tri-phosphate (ATP) is a major energy substrate and involved in such physiological functions as sleep and regulation of inflammation [2]. ATP is found in all nucleated cells in the body including neurons and glia. ATP is co-released into the extracellular space with neurotransmitters such as GABA and glutamate. Overall, ATP levels in the brain are reduced during sleep compared to wakefulness [179]. ATP agonists enhance sleep and SWA, while ATP antagonists inhibit sleep and SWA [130]. Moreover, enhanced neural activity increases ATP production and extracellular ATP release, which may contribute to oxidative phosphorylation enhancement after sleep deprivation [180]. Collectively, these data indicate that enhanced neuronal activity releases ATP that then traverses the membrane and enters the extracellular space, which leads to downstream mechanisms that may initiate sleep, including enhancement of extracellular adenosine levels. Extracellular ATP directly binds to purinergic type 2 receptors on neurons and glia throughout the brain, which in turn activates many sleep-enhancing molecules including IL-1β, TNF-α, brain-derived neurotrophic factor (BDNF), cyclic AMP, phospholipase C, arachidonic acid, and NO [2]. Mice lacking the purine type 2 X7 (P2X7) receptor have attenuated NREMS and SWA responses to sleep deprivation [143]. Furthermore, P2X7 receptors are down-regulated after sleep deprivation in the somatosensory cortex and hypothalamus of rats. This evidence collectively indicates that enhanced waking activity enhances extracellular ATP and, through the P2X7 receptor, modulate sleep.

ATP is metabolized to adenosine diphosphate (ADP) and adenosine monophosphate (AMP) by ectonucleoside diphosphohydrolase, also known as CD39 [2]. Cyclic AMP influences glial and neuronal sleep regulation, also suggesting that molecules downstream of ATP are involved in regulating sleep [181]. AMP is metabolized to adenosine by the rate limiting enzyme ecto-5′-nucleotidase, also known as CD73 [182]. Mice lacking CD73 have attenuated SWA and sleep responses to enhanced waking activity, suggesting that the metabolism of the ATP bi-product adenosine is involved in sleep regulation.

### 10.2. Adenosine

The purine adenosine is produced by most cells, including neurons and glia of the CNS [21]. Adenosine functions to regulate inflammation, vasodilation, cerebral blood flow, and sleep [21,183]. Wilhelm Feldberg and Stephen Sherwood first demonstrated the sleep-enhancing effects of adenosine after direct administration into the left ventricle of cats [184]. Thereafter, both central and systemic application of adenosine and adenosine A1 receptor agonists were found to enhance sleep [185]. The widely used stimulant caffeine promotes wakefulness, in part, by antagonizing adenosine receptors [21]. Pioneering microdialysis studies by McCarley and colleagues found that adenosine played a major role in the induction of sleep, particularly by means of inhibition of wake-active neurons in the basal forebrain and cortex [186]. Further studies by this group found that, after sleep deprivation, adenosine A1 receptors are up-regulated, and their density enhanced [187,188]. Other investigators reported that adenosine A2a receptor agonists enhance NREMS and REMS [189]. Adenosine A2a receptor effects on sleep are mediated, in part, through action on GABAergic neurons in the hypothalamus, particularly the VLPO [190,191]. Nevertheless, A1 receptor knockout mice had similar EEG responses after sleep deprivation compared to wild type animals [192]. Adenosine A2a receptor knockout mice, however, demonstrate attenuated homeostatic sleep responses to sleep deprivation and caffeine [193,194]. Evidence also indicates that adenosine inhibits basal forebrain cholinergic neurons *in vivo* and *in vitro* [195,196], attenuating the wake-promoting actions of acetylcholine.

### 10.3. Lactate

Lactate is produced from the breakdown of glucose [197]. Glucose is the preferred energy source for most cells including neurons, although neurons can also utilize lactate, in part, through a lactate shuttle involving glia. Nevertheless, the physiological relevance of lactate fluctuations after neuronal stimulation remains controversial. Glucose is enhanced in the brain during sleep relative to wakefulness, suggesting an enhanced demand of glucose as an energy source during sleep [198]. Since glucose utilization during NREMS is reduced compared to waking, and lactate is generated from glycogen breakdown [199], lactate has been hypothesized to influence neuronal activity involved in sleep/wake regulation. Furthermore, ATP levels are reduced in the brain during sleep after wakefulness suggesting that a shift from aerobic glycolysis to oxidative phosphorylation occurs during sleep [179]. Also, genes related to the lactate shuttle and glycogen, such as glucose transporter 1 (GLUT1), are altered after sleep deprivation [200,201]. Indeed, cerebral lactate concentrations in the cerebral cortex are elevated with wakefulness and REMS, and decline with NREMS [202]. Moreover, cortical theta band activity is positively correlated with extracellular release of lactate in the rat basal forebrain [203], and optogenetic stimulation of cortical pyramidal cells induces a reduction in extracellular lactate levels that correspond with reductions in slow wave oscillations, again suggesting a link between sleep-related lactate levels and SWA [204].

### 10.4. Waste clearance

The formation of reactive oxygen species and detrimental substances that accumulate during enhanced activity, such as seen during wakefulness, can potentially damage cells and tissues [205]. In 1968, Földi and colleagues first suggested that perivascular spaces act as lymphatics in the brain [206]. This system, referred to as glymphatic system, is driven by cerebrovascular pulsation and depends on astroglial water channels that line perivascular channels. Exciting recent investigations have demonstrated that glymphatic drainage is associated with sleep-wake states, showing an increased clearance of molecular waste during sleep compared to wake [207]. Consequently, it is has been hypothesized that a function of sleep is to enhance the clearance of metabolic waste products that accumulate during wakefulness.

## 11. Cellular Pathways

Various cellular pathways are activated or inhibited by sleep regulatory molecules, and many of these molecules are reciprocally activated or inhibited by these same pathways [2]. Cellular pathways that regulate sleep also modulate inflammation, neurotransmission, and ionic changes within neurons and glia.

### 11.1. Nuclear factor-kappa B

Nuclear factor-kappa B (NF-κB) is a transcription factor found in all nucleated cells [199]. In response to cytokines, pathogens, or neurotransmitter activity, NF-κB is activated and promotes the transcription of numerous genes that regulate sleep [2]. NF-κB is regulated by different subunits including p50, p52, p65, RelA, RelB, and c-REL [208]. NF-κB dimer subunits in the cytoplasm are inhibited by inhibitors of κB (IκB), and activation of NF-κB occurs when IκB kinase degrades the IκB protein. This allows the NF-κB complex to enter the nucleus and trigger the expression of specific genes, including humoral factors that regulate sleep such as IL-1β and TNF-α, which reciprocally also activate NF-κB.

A diurnal rhythm of NF-κB activation in the cortex corresponds with sleep propensity [208]. After sleep deprivation, enhanced NF-κB levels have been documented in the cortex and lateral hypothalamus of mice and rats, respectively [209,210]. Additionally, in rats, enhanced NF-κB nuclear translocation was shown after sleep deprivation in basal forebrain cholinergic neurons that express adenosine A1 receptors [211,212]. Peptidergic inhibition of NF-κB activation attenuated both spontaneous sleep and sleep enhancement produced by IL-1β administration [213]. The use of the inhibitor peptide, SN50, prior to 6 hours of sleep deprivation decreased SWA during recovery sleep in rats [212]. In addition, mice lacking the NF-κB subunit p50 have an enhanced NREMS response to LPS, as well as more fragmented sleep after influenza infection. Additionally, mice lacking the NF-κB p50 subunit have altered expression of adenosine A1 and A2a receptors, which might contribute, in part, to enhanced NREMS and REMS during spontaneous sleep reported in these mice [214,215]. In summary, NF-κB appears to play a substantial role in modulating energy- and inflammatory-related substances that have been shown to modulate sleep.

### 11.2. Nitric oxide

Nitric oxide (NO) functions, in part, to modulate vasohemodynamics, inflammation, and sleep [216–217]. Nitric oxide (NO) is produced from arginine in the presence of nicotinamide adenine dinucleotide phosphate and dioxygen. This reaction is catalyzed by nitric oxide synthase (NOS) [216]. Many cells produce NO in the CNS, including endothelia, neurons, and glia. There are three forms of NOS: neuronal NOS (nNOS), endothelial NOS(eNOS), and inducible NOS(iNOS).

Much evidence indicates that NO is a key molecule that regulates both sleep and SWA. Administration of the NO inhibitor, L-nitro-arginine methyl ester (L-NAME), attenuated NREMS and REMS responses to sleep deprivation in both rats and rabbits [217–219]. Conversely, the NO donors 3-morpholinosydnonimine (SIN-1) and S-nitroso-N-acel-D, L-penicillamine (SNAP) enhance NREMS in rats [220]. Mice lacking iNOS have reduced NREMS during the active (dark) period [221], and mice lacking nNOS have an attenuated SWA response to sleep deprivation [125]. The enhanced NREMS response due to influenza is attenuated in both nNOS and iNOS KO mice [222]. After 11 h of sleep deprivation, iNOS and NO levels (sampled by microdialysis) are elevated in the basal forebrain and frontal cortex of rats, followed by enhanced adenosine levels. In addition, iNOS and c-fos immunoreactivity is enhanced in the basal forebrain and frontal cortex in wake-active neurons, further suggesting that iNOS may play a role in vigilance state regulation [223,224]. Furthermore, systemic injections of TNF-α did not enhance sleep in nNOS knockout mice [225]. Recently, select GABAergic neurons in the cortex that express nNOS were found to be sleep-active and associated with changes in SWA responses to acute sleep loss [226].

### 11.3. Inflammasomes

Inflammasomes are protein complexes that develop in response to exposure to various biological, chemical, and pathogenic stimuli [227]. Inflammasomes are present in many cells types including neurons, microglia, perivascular macrophages, and astrocytes [227,228]. Pathogen associated molecular patterns (PAMPs)(e.g., LPS, muramyl dipeptides, influenza) acting through their respective pattern recognition receptors (PRRs) [e.g., toll-like receptor 4 (TLR4) for LPS), as well as extracellular adenosine tri-phosphate (ATP) release by means of purine type 2 receptors (P2Rs), activate inflammasomes [227]. There are many P2Rs that are implicated in inflammasome activation including P2X7R, P2X4R, P2Y1, and P2Y12 [229]. A main component of the inflammasome is the nucleotide oligomerization domain-like receptor (NLR) that recognizes the activation of PAMPs to their PRR, inducing intracellular formation of the inflammasome protein complex [227]. Inflammasomes, including nucleotide-binding domain, leucine rich family pyrin containing 3 (NLRP3), are activated by unique substances, chemicals, or PAMPs through their PRRs. The NLRP3-inflammasome is the most well-characterized of the inflammasomes. Inflammasomes activate the enzyme caspase-1, which cleaves the pro-forms of IL-1β and IL-18 into their mature active forms, resultantly inducing inflammation. In addition, the apoptosis-associated speck-like protein containing a C-terminal caspase-recruitment domain (CARD) (ASC) acts as an adaptor protein that gathers pro-caspase-1 into the inflammasome complex, and is required for inflammasome formation under most conditions. Extracellular ATP and LPS (via the P2Rs including the P2X7R and TLR4, respectively) activate the NLRP3 inflammasome. As previously mentioned, evidence suggests that extracellular ATP, the P2X7R, and LPS enhance sleep and IL-1β in the brain [2,143]. Minocycline is a drug that inhibits caspase-1 and IL-1β, and has been shown to attenuate sleep in mice, further suggesting that inflammasomes function, in part, to regulate sleep [171,230]. Moreover, inhibiting caspase-1 pharmacologically inhibits sleep in rats [231]. Mice lacking NLRP3 have reduced sleep and SWA responses to enhanced waking activity and LPS (Zielinski et al., unpublished findings). Consequently, inflammasomes may provide the pathway by which local environmental change is detected in the brain, including that of energy-related molecules and pathogens and their components, in turn affecting sleep regulation.

### 11.4. Stress Pathways

Stress is typically defined as a response to physiological demands placed on an organism [232]. Classically, the autonomic sympathetic-adrenal system and the hypothalamic-pituitary-adrenal axis are two neural circuits that influence the organismic responses to stress. Sympathetic activation of the nervous system releases norepinephrine from the sympathetic nerve terminals, as well as secretion of epinephrine from the adrenal medulla. Stress induces corticotrophin-releasing hormone (CRH) from the hypothalamus, which in turn induces the release of adrenocorticotropic hormone (ACTH) from the pituitary. ACTH then promotes glucocorticoids release from the adrenal cortex.

Stress-related molecules tend to exhibit diurnal patterns [233]. A rhythm of glucocorticoids has been shown, as levels are highest toward the end of the inactive (light) period in rodents immediately preceding the most active part of the day, the beginning of the active (dark) period. Some studies have demonstrated modest enhancement in glucocorticoids after acute or chronic sleep loss, but others have reported no change in glucocorticoid levels [234–238]. Compared to other stressors, glucocorticoid responses are quite subdued, although changes in neuronal plasticity, neurogenesis, and mood can occur with minute alterations in stress hormones [238]. However, ACTH and CRH responses are attenuated in sleep-restricted rats compared to controls [235,239]. Interestingly, the wake-promoting effects of CRH might occur, in part, through interactions with IL-1β [240]. CRH can inhibit IL-1β, and central injections of CRH have been shown to increase sleep latency and attenuate NREMS in rabbits [230]. This effect may occur, in part, through CRH induction of glucocorticoids which have well-established anti-inflammatory actions.

## 12. Local Sleep

Sleep occurs not just within an entire organism (globally) but within localized areas and networks of cells (locally) [2]. Evidence for “local sleep” is found in cases of individuals with parasomnias, who can walk, eat, or have sex but are not entirely conscious [241–243]. An intriguing investigation in epileptic patients with cortical indwelling electrodes demonstrated “local” sleep, in which, during sleep, NREMS characteristics such as SWA and spindles were localized to select regions of the cortex [244]. It was therefore suggested that, as wake is extended by such means as sleep deprivation, sleep-like local activity may intrude into the wake EEG as select neural circuitry goes “offline”. Indeed, Vyazovskiy, Tononi, and colleagues then reported that in the rat, during extended periods of wakefulness, select cortical neurons and related circuitry enter an offline sleep-like state accompanied by SWA in localized cortical regions [245]. This “local sleep” activity increases in incidence as wakefulness was extended. Therefore, although the animal was described by global EEG recordings as awake, localized cortical EEG and neuronal activity recordings indicated that local neuronal populations in the cortex exhibited sleep-like activity. Other evidence of “local sleep” includes studies in which stimulating rodent vibrissae (i.e., whiskers) increases SWA in respective cortical columns, as well as enhances the activity of IL-1β and TNF-α immunoreactive cells [246–248]. Application of TNF-α locally onto one cortical hemisphere enhances SWA, and activates c-Fos and IL-1β in the respective hemisphere, compared to vehicle control treatment of the contralateral hemisphere [249,250]. Recently, Krueger and colleagues developed a novel methodology demonstrating a sleep-like state in a mixed glia and neuron cell culture network [251,252], which was more extensively developed by Tafti and colleagues [253]. Of particular importance, this method was able to document cellular signaling changes after the addition of neurotransmitters, resembling responses observed in mammals during wakefulness.

## 13. Conclusion

In summary, we now understand that sleep is regulated globally, regionally, and locally, by means of both molecular and cellular mechanisms. Redundancy in the molecules, cells, and neural circuitry regulating sleep suggest that sleep plays a crucial and protective physiological role, yet such redundancy also renders elusive exact determination of the function(s) of sleep and the regulatory mechanisms involved. Nevertheless, recent basic science technologies are rapidly advancing, further expanding our understanding of the function(s)of sleep.

## Figures and Tables

**Table 1:** Gross physiological characteristics of Wakefulness, NREMS and REMS.

Wakefulness	NREMS	REMS
Enhanced movement activity	Reduced movement activity	Reduced movement activity (cataplasia)
Opened eyes	Closed eyes	Closed eyes (rapid-eye movements)
Enhanced responsiveness to external stimuli	Reduced responsiveness to external stimuli	Reduced responsiveness to external stimuli
Variable body position	Recumbent body position	Recumbent body position
Variable breathing rate	Regular breathing rate	Variable breathing rate

**Table 2:** Electroencephalogram power bands.

Frequency band	Frequency range	Dominant sleep stage	Dominant brain areas involved in regulation	Associated functions
Delta	0.5–4.0 Hz	NREMS	Cortex, thalamus, hypothalamus, basal forebrain	Slow-wave sleep, memory consolidation, synaptic homeostasis, health
Theta	4.0–9.0 Hz	Wake and REMS	Hippocampus, medial septum, brainstem, hypothalamus	Cognition, memory consolidation, movement activity, REMS, development
Alpha	9.0–15.0 Hz	Wake	Thalamus, cortex including the occipital lobe	Visual activity and cognition
Beta	15.0–30.0 Hz	Wake	Cortex	Anxious thinking, alertness and critical reasoning
Gamma	>30.0 Hz	Wake and REMS	Neocortex, cortex and basal forebrain	Sensory processing, memory, cognition
